# Yaws Prevalence, Lessons from the Field and the Way Forward towards Yaws Eradication in Ghana

**DOI:** 10.1155/2014/910937

**Published:** 2014-12-31

**Authors:** Patrick Agana-Nsiire, Ekow Kaitoo, Emmanuel Erasmus Akurugu Agongo, George Bonsu, Sadik Kyei-Faried, Kwame Amponsa-Achiano, Kofi Ahmed, Ebenezer Appiah-Denkyira, Kingsley Asiedu, Joseph Amankwa, Frank Adae Bonsu

**Affiliations:** ^1^National Yaws Eradication Program, Disease Control and Prevention Department, Korle Bu, Accra, Ghana; ^2^East Akyem Municipal Health Directorate, Ghana Health Service, Kibi, Ghana; ^3^Policy, Planning, Monitoring and Evaluation Division, Ghana Health Service, Accra, Ghana; ^4^Expanded Program on Immunization, Disease Control and Prevention Department, Korle Bu, Accra, Ghana; ^5^Disease Control and Prevention Department, Ghana Health Service, Accra, Ghana; ^6^National Yaws Eradication Program, Ghana; ^7^Ghana Health Service Headquarters, Accra, Ghana; ^8^WHO Headquarters, Geneva, Switzerland; ^9^Public Health Division, Ghana Health Service, Accra, Ghana; ^10^National Tuberculosis Control Program, Disease Control and Prevention Department, Korle Bu, Accra, Ghana

## Abstract

Despite past WHO/UNICEF led global yaws eradication efforts, the disease seems to persist. The true burden is however not known for comprehensive action. Ghana's data showed significant increase in notified cases since the 1970s. Recognizing limitations in routine data, we carried out a yaws treatment survey in 2008 in three purposively selected districts to establish the prevalence and learn lessons for renewed action. Of 208,413 school children examined, 4,006 were suspected yaws cases (prevalence 1.92 (95% CI: 1.86–1.98) percent). Of 547 schools surveyed, 13% had prevalence between 5% and 10% while 3% had prevalence above 10%. The highest school prevalence was 19.5%. Half of the schools had cases. The large sample allowed aggregating the school results by administrative levels. The lowest aggregated prevalences of 0.23%, 0.40%, and 0.64% were in the urban sub-districts of Asamankese, Oda, and Achiase, respectively, while the highest of 8.61%, 3.69%, and 1.4% were in rural Akroso, Mepom, and Aperade, respectively. In conclusion, the prevalence of yaws is high in some schools in rural, hard-to-reach areas of Ghana. Considering past global eradication efforts, our findings suggest yaws may be resurging for which programmatic action is needed.

## 1. Introduction

**Figure 1:**
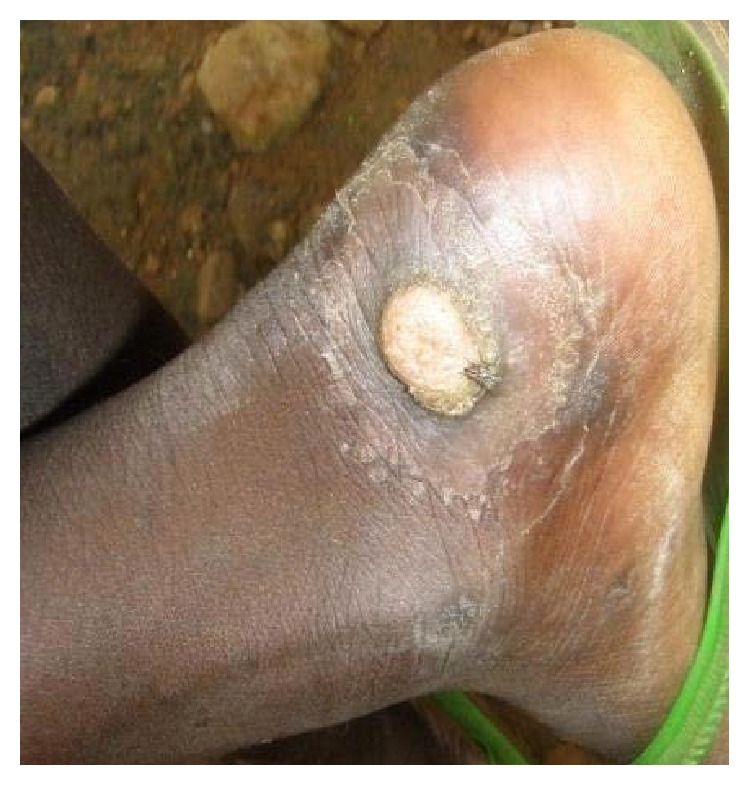
Papilloma.

Yaws, a neglected tropical disease, is a bacterial disease of the skin, cartilage, and bones caused by* Treponema pallidum* subsp.* pertenue*. Symptoms and signs include typical papillomas (Figure 1), other varied skin rashes, and mild joint and bone pains and swellings affecting the finger bones (dactylitis), the long bones, and bone and cartilage of the face. If left untreated, about 5% of early yaws cases progress to late stage deformities like gangosa, gondou, polydactylitis, sabre tibia, or scars and contractures.

Due to the high morbidity of treponemal diseases and the ease with which they responded to treatment with single dose depot penicillin, a 1949 World Health Assembly (WHA) resolution [1, 2] gave birth to the yaws eradication effort. In Ghana (then Gold Coast), according to the Colonial Reports [3], yaws ranked among the top diseases like malaria, guinea worm, and onchocerciasis. A World Health Assembly resolution (WHA2.36) mandated the control of the treponemal infections in 1949. Based on this resolution and encouraged by the good results produced by single dose depot penicillin given in mass treatment against yaws in a number of endemic countries [4], WHO and UNICEF launched a yaws eradication program between 1952 and 1965 as reported by Perine and others [5]. The main strategy was mass treatment of cases and contacts with penicillin aluminum monostearate in oil (PAM). Though it greatly reduced the number of cases, global eradication of yaws was not achieved. Some individual countries and WHO regional groupings made further efforts at yaws elimination within their borders with varying degrees of success. Renewed efforts in Ghana from 1981 to 1983 as reported by Agadzi and others [6] combined treatment for yaws with yellow fever and tetanus immunization. These efforts were short lived, and yaws activities, integrated into routine services, have since received little attention in the face of competing priorities. The noticeable increase in yaws case notification observed in Ghana (from routine and haphazard case searches and treatment) when the global eradication effort stopped suggested possible yaws resurgence. Given the limitations of the data then available, the newly organized National Yaws Eradication Program (2008) carried out a pilot treatment survey in the same year, combining case searches with treatment. The objectives of the study were to estimate yaws prevalence in school children in the selected districts and draw implementation lessons for national program action.

## 2. Materials and Methods

### 2.1. Study Design and Period

This was a prevalence treatment survey conducted from October to December 2008. The period of the survey was purely incidental though October–December is a minor rainy season in Ghana, relatively dry when yaws prevalence is generally expected to be lower as Hill reports [7]. The survey was integrated as part of school health program activities of screening for health and other related conditions.

### 2.2. Sampling

**Figure 2:**
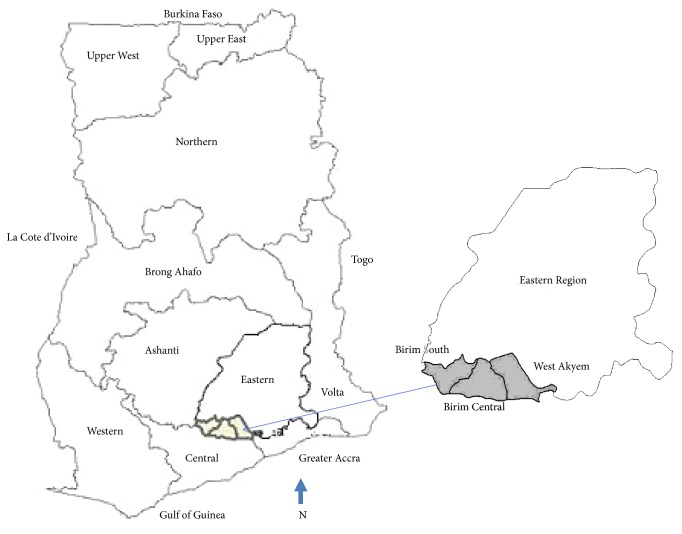
Pilot districts in the Eastern Region of Ghana.

We purposively sampled three districts in the Eastern Region of Ghana (Figure 2) because of their high yaws case notification in the country (16,319 cases between 2005 and 2007). All basic schools were included and all children present on days of visit were screened.

### 2.3. Planning and Social Mobilization

Planning, which lasted several weeks, involved national, regional, and selected district and subdistrict health and education staff, as well as community agents. We employed widespread social mobilization techniques such as use of health workers and local opinion and traditional leaders and local public address systems and sent letters to schools. This was principally to engender community support for the intervention.

### 2.4. Training of Health Staff

We organized a cascaded training from regional to district and subdistrict levels with each training lasting one day. Training aids included picture cards and live cases for demonstration. We trained participants on case identification, adverse drug reaction management, data collection, and tools as well as data management. Teachers helped to mobilize the pupils but were not deliberately trained for it.

### 2.5. Eligibility and Enrolment

All children aged 15 years and below who were present in school during the study period were eligible to be enrolled into the study.

### 2.6. Cases Ascertainment and Recording

Health workers physically examined all children and tallied those with lesions under* Yaws* if yaws was suspected or under* Other ulcers/lesions* in the tally sheet. The clinical case definition of yaws was as follows: a papilloma, an ulcer with raised edges, tender bone and joint swellings, and skin papules and macules suspected by the trained field technicians to be yaws. Where the communities were entered to trace and treat contacts and community cases, these were not added in calculating the school based prevalence. All suspected cases and their identified contacts were treated with intramuscular injections of benzathine penicillin and adverse drug reactions (ADRs) were observed and managed appropriately. In the treatment of contacts, adults were included.

### 2.7. Data Management

We entered data in a customized Microsoft excel template and analyzed it in excel (Microsoft Corp.) and Stata version 10.2 software (Stata Corp. Texas, USA). Data were analyzed in aggregated fashion with no link to individual records to ensure confidentiality. Prevalence was estimated by school, by subdistrict, and by district and presented as point estimates with 95% confidence bands using exact binomial methods in Stata version 10.2 software. The levels of endemicity corresponded to levels proposed for control purposes by Hackett and Guthe [8] as follows: hypoendemic (prevalence <5%); mesoendemic (prevalence 5–10%); hyperendemic (prevalence >10%). The school prevalences were however proxy levels for decision taking as they were not based on the total population.

### 2.8. Ethical Considerations

Yaws treatment with penicillin is an ongoing service in Ghana. The pilot was therefore carried out as an integral part of routine program services. No special ethical clearance was therefore required. The usual social mobilization for a mass exercise was carried out involving the parents, community leaders, and education authorities to enter the schools at their convenience and for teachers to support the exercise. The Eastern Regional Health Directorate was notified of the specific activity in three of their districts and took part in the training and supervision. Data collected was totally anonymous except for the few adverse drug reactions which were followed up on by the Ghana Food and Drugs Authority office which monitors drug and immunization events in Ghana. Children and community contacts were however not compelled if they refused the injections, but, following pretreatment counseling, no child diagnosed with skin lesions refused treatment. Adequate measures were taken to address any adverse reaction from the treatment.

## 3. Results and Discussion

### 3.1. Results

A total of 208,413 children aged about 15 years and below were examined of which 4,006 were cases, giving an overall school based prevalence of 1.9% (95% CI: 1.9–2.0). In terms of schools surveyed, a total of 547 basic schools were covered (Table 1). Of these 51% had no cases and required no treatment. Thirty-four (95% CI 30–38) percent had prevalence less than 5%; 11 (95% CI 9–14) percent had prevalence 5–10% while three (95% CI: 2–5) percent had prevalence greater than 10%. Table 1 shows distribution of schools surveyed by level of endemicity.  Table 2 summarizes the key findings. As this was a large sample of school children, data was also pooled at district and subdistrict levels for analysis (Tables 2 and 3, resp.). Individual district level prevalences were 0.98 (95% CI: 0.86–1.11) percent for Birim South, 1.12 (95% CI: 1.03–1.21) percent for West Akyem, and 2.41 (95% CI: 2.32–2.49) percent for Birim Central. Subdistrict level prevalences ranged from 0.23 to 8.61. The lowest prevalences were found in the urban areas (e.g., 0.23% in Asamankese, capital of West Akyem Municipality, and 0.40% in Oda, capital of Birim Central Municipality, resp.). The highest prevalences occurred in the rural subdistricts: 8.61% in the difficult-to-access subdistrict of Akroso in Birim Central and 3.69% in rural Mepom in West Akyem. Individual school prevalences ranged from 0% to 19.5% in West Akyem. Cases were found in half the schools in the three districts (49%).

A total of 71,152 persons were treated by giving benzathine penicillin intramuscularly on the upper outer quadrant of the buttock. Big girls and adult cases and contacts identified on contact tracing to the communities were given doses on the lateral aspects of their thighs. Cases aged 10 years and above were treated with the full vials of 1.2 mega units while cases less than 10 years and contacts 10 years and above were treated with 0.6 mega units. Contacts less than 10 years were given 0.3 mega units. The 71,152 persons treated comprised 4,950 cases (this included cases found in the communities during contact tracing) and 67,146 contacts. A small number of household adult contacts (less than 1 in 10) refused to take the injection. In one school in Birim Central Municipality, 184 cases had been treated during exploratory visits earlier in the year (March 2008) and their names were entered into a register that was kept in the school. Five of these cases were still found with yaws-like lesions during the pilot treatment survey in October 2008 and were retreated. In addition, 86 new cases were found and treated in the same school. One probable yaws deformity involving bones of the left leg in an old yaws case was seen among the 621 suspected cases in one subdistrict of West Akyem. Overall, there were six mild ADRs among all persons treated, giving an ADR rate of 8.4 (95% CI 3.1, 18.4) per 100,000. One was an injection abscess in a nine-year-old boy that resolved on amoxicillin. The rest were skin itching and urticarial rashes that resolved quickly.

### 3.2. Discussions

The overall prevalence of 1.92% compares with 1.82% found in the same year during rapid school surveys in the Western Region of Ghana which has similar climatic conditions as the study area (unpublished). Such aggregate prevalence however hides localized high burden of disease as shown by the school with prevalence of 19.5%. Subdistricts may also have low aggregate prevalences thus hiding satellite communities in that subdistrict with high prevalence because their population is small and vice versa. In August 2008, a month before the commencement of the pilot activities, the three districts routinely reported a total of 135 cases. Assuming a monthly routine case notification rate of 135 cases, this would be only 3% of the 4006 cases detected by active case search during the 2-week survey. This means routine notification misses 97% of cases. This is reemphasized by the finding that all the 170 districts in the country reported 28,080 cases throughout all of 2008 and 4006 cases (14%) of these were found in just 2 weeks among school children in three districts. The findings made must however take into account obvious limitations. Cases were diagnosed clinically with no laboratory confirmation. Also, a one-day capacity building for health workers to detect cases clinically may not be sufficient and therefore could influence diagnosis. Following these studies in 2008, public education on yaws was intensified with the hope of detecting several more thousands of cases through nationwide campaigns and planned community treatment surveys by districts and subdistricts. The needed resources did not come however to support this plan, emphasizing the real neglect of the yaws disease. The annual number of cases detected and treated therefore rose from 28,080 in 2008 to 36,326 in 2009 and then fell to 19,917 in 2013 as reported in the Ghana National Yaws Eradication Program Annual Report 2013. This fall thus partly reflects the impact of eradication activities in the limited areas reached but excludes the larger numbers that would have been detected with more resources. The contact: case ratio (i.e., number of contacts treated per case of yaws detected) which is an indicator of the effectiveness of eradication activities however climbed progressively from 3.5 in 2008 to 10.8 in 2013. This means in the limited areas covered, trained personnel were more conscious and effective in tracing and treating contacts which is the backbone of yaws eradication activities.

Studies and reports from other countries give similar findings as the Ghana study results. In Cameroon, for example, Coldiron and others [9] did a treatment survey among 6000 children in 2 districts. The clinical cases were 485 children and those serologically positive were 183 children or 2.9% (95% CI 2.6–3.4). In another study by Touré and others [10], all health districts in Cote d'Ivoire reportedly notified yaws at an incidence rate of 0.58% in 2000 while a follow-up cross-sectional survey revealed a clinical prevalence of 5%. A 1985 survey by Touré [11] of three districts in Togo, some of which border Ghana, established clinical prevalences of 1.0–3.9%. In a study by Gerstl and others [12] in the Republic of Congo, an overall prevalence of 4.7% (95% CI 3.4–6.0) was observed using serological confirmation of clinical lesions. Among a total of 14 lots, two had prevalence over 10% while three were less than 5. Studies in Vanuatu [13] and the Solomon Islands [14] give similar findings. The true extent of the current yaws problem is however not fully determined globally. These isolated studies however indicate definitely that yaws is not eradicated yet and may be resurging. It is probably safe to say that the current yaws situation in countries is a result of unfinished business of the global eradication effort of the 1950s and 1960s when the program was devolved by WHO and UNICEF to countries. If yaws exists as a zoonosis in other primates like chimpanzees and gorillas as reported by Harper and others [15] and Centurion-Lara and others [16], there is the possibility of cross species transmission especially given the close contact between wild life conservation workers and the culture of killing and eating bush meat in parts of the world where yaws is endemic. This could also explain yaws reemergence and equally importantly has bearing on yaws eradication goals and strategies. But as there are no reports of such cross transmission in the literature, assuming different strains are at work, yaws eradication from the human population is still a practical possibility for global consideration.

A number of lessons may be drawn from this treatment survey for program purposes. It is possible to combine treatment in yaws surveys or vice versa as may often be necessary in resource constrained areas to maximize efficiency. The differing levels of endemicity in schools possibly mirror the differing characteristics of their communities that predispose them to different intensities of yaws transmission and infection. Although these require further studies, the effect of urbanization and deprivation on yaws was clearly shown (Table 3). Urbanization here refers to all district capitals and similar towns with basic amenities like schools, potable water, and easy geographical access to health facilities. The focal occurrence of yaws was also demonstrated by half the schools having clinical cases and the other half having no cases. Where focality of yaws involves hard-to-reach areas, this makes eradication activities more difficult. There are many such areas and situations in Ghana involving seasonally inaccessible communities, temporary farm huts, and nomadic herdsmen. The five retreated yaws-like cases in the one school in Birim Central Municipality, where 184 cases were treated earlier in the year, raise issues of possible further research interest. Mitjà and others [17] in a recent study have indicated the possibility of* Haemophilus ducreyi* ulcers presenting like yaws ulcers. Resistance to treatment in this geographical area should also be considered but this was not done for the 5 cases because PCR for yaws was and still is unavailable and exporting samples outside was not considered for logistic reasons. The five cases were therefore not followed up with further studies to clarify their status and they could well be reinfections as yaws does not confer permanent immunity. Additionally, the finding of 86 new cases in the same school emphasizes the importance of contacts and latent yaws cases in eradication programs. Contacts are incubators and latent cases are established infections with germs in the blood but no overt lesions. They, however, show as overt and contagious lesions from time to time. Both contacts and latent cases are missed during clinical screening. The one probable yaws deformity involving bones of the left leg in one subdistrict of West Akyem shows that late yaws lesions with deformities may still be occurring and that presents a case of health service neglect in rural communities. In the Ghana azithromycin efficacy study (yet unpublished), early tibia bowing and forearm bone abnormalities were seen in young boys and girls which, luckily, resolved after treatment. Facial deformities (gangosa) in 2 young children, both 13 years old, were found in the same area in 2013, dating back about three years, from the history given by the guardians. TPPA which was available for another pilot in 2013 was done on these 2 boys and turned out strongly reactive for both. Dilutions were not done. ADRs with penicillin vary from mild to severe with occasional fatalities. Practically any organ can react to penicillin but the commonest are skin reactions including the pain of injections. In this treatment survey, the overall ADR rate of 8.4 per 100,000 (0.0084%) suggests a high benzathine penicillin safety profile compared to the observed ADR rates of 2%–15% from full course treatment regimens given to out- and inpatients as quoted by Ditto [18], Jick [19], or Mendelson [20]. This may be a peculiarity of the study population but is more likely because of the single doses given in programmatic situations. Difficulties relating to use of injections in a mass setting included refusal by a few contacts, both children and adults. Some children squeezed their buttocks making injection difficult and risky. In these cases the injections were given on the thigh, still with some difficulty. The adult contacts shied away from exposing their buttocks and were given the injections on the thighs as well. These reactions do emphasize, however, the challenges posed by injection use in yaws eradication programs. The involvement of teachers well before such programs may go a long way to minimize such fears and reactions by pupils. Last but not least, the high underreporting of yaws cases probably suggests that a routine case detection approach only may not adequately address eradication objectives.

Following recent studies on the use of single-dose azithromycin for treating yaws in Ghana (unpublished) and Papua New Guinea published by Mitjà et al. [21] Ghana must rapidly review, revise, and consider adapting to current WHO recommended single dose of Oral Azithromycin for the treatment of yaws [14]. This form of treatment would ease the challenges with the use of injection benzathine penicillin in mass treatment campaigns. In 2013, a rapid dual nontreponemal and treponemal point-of-care syphilis test (DPP^®^ Syphilis Screen & Confirm Assay Chembio, USA) was evaluated for yaws in four countries including Ghana. Published results for Papua New Guinea by Harper et al. [15] are promising and can now be incorporated into yaws activities in the field to confirm cases on the spot and improve surveillance. PCR technology is also available to accurately diagnose yaws and identify any resistance mutations to azithromycin. Any future studies should therefore provide adequately for employing such new technologies to confirm complicated nonhealing cases of yaws and monitor resistance. The possibility of* Haemophilus ducreyi* causing yaws-like ulcers is of epidemiological and clinical significance and needs to be further studied.

## 4. Conclusions

Yaws is still prevalent among children in some schools in Ghana. It is a source of concern especially in hard-to-reach communities, considering previous programmatic attempts at eradication. The observed high prevalence of more than 10% in 3% of schools surveyed may be an early warning of yaws resurgence and, therefore, there is urgent need to take programmatic action against yaws considering the new technologies available. Mass treatment for yaws is feasible and implementable with good planning in integrated health services. The fact that a number of countries have eliminated yaws from their borders suggests the possibility of eradicating the disease from the human population even if it exists in some wild primates as indicated by some authors. This study, carried out as part of a service delivery package, seeks also to reemphasize that we can minimize lost opportunities in programs like yaws eradication by providing service while simultaneously collecting data to help inform us to prepare better for more services tomorrow.

## Figures and Tables

**Table 1:** Distribution of schools by level of endemicity.

District	Number of schools surveyed	Number of (%) schools prev. = 0 endemic	Number of (%) schools prev. <5%	Number of (%) schools prev. 5–10%	Number of (%) schools prev. >10%
Birim Central	204	104 (51.0)	50 (24.5)	39 (19.1)	11 (5.4)
Birim South	81	35 (43.2)	41 (50.6)	5 (6.2)	0 (0.0)
West Akyem	262	142 (54.2)	96 (36.6)	18 (6.9)	6 (2.3)
Total	**547 **	**281 (51.4) **	**187 (34.2) **	**62 (11.3) **	**17 (3.1) **

**Table 2:** District level and overall aggregated results of school-based yaws surveys.

District	Number of children examined	Number of suspected yaws cases	Prevalence of yaws % (95% CI)
Birim Central	132292	3185	2.4 (2.3–2.5)
Birim South	23070	227	1.0 (0.9–1.1)
West Akyem	53051	594	1.1 (1.0–1.2)
Total	**208413**	**4006**	**1.92 (1.86–1.98)**

**Table 3:** Treatment survey results aggregated by subdistricts.

District	Subdistrict	Number of children examined	Number of yaws cases	Yaws prevalence
Birim Central Municipality	Akroso^R^	30068	2589	8.61
	Asene^R^	16031	183	1.14
	Manso^U^	36080	214	0.59
	Oda^U^	50113	199	0.4

Birim South District	Achiase^U^	10466	67	0.64
	Aperade^R^	4343	61	1.4
	Akim Swedru^U^	8261	99	1.2

West Akyem Municipality	Abamkrom^R^	5555	102	1.84
	Adeiso^R^	11750	244	2.08
	Asamankese^U^	17601	40	0.23
	Brekumanso^R^	4005	44	1.1
	Mepom^R^	2843	105	3.69
	Osenase^U^	11297	59	0.52

^R^Rural; ^U^Urban. The rural/urban classification was qualitative. All district capitals and towns as big as or with similar facilities to district capitals were considered urban, otherwise rural. Akim Swedru subdistrict, whose main town, Akim Swedru, is also the district capital, had many rural satellite communities as part of the health subdistrict catchment area and hence the high prevalence of that subdistrict.
